# Gut microbiota-derived small molecules, peptides, and proteins in GPCR signaling: current evidence for β-arrestin involvement

**DOI:** 10.3389/fmicb.2026.1979411

**Published:** 2026-10-08

**Authors:** Yushan Hu, Yiru Liu, Haiyan Yao, Siteng Wang, Yice Gao, Ruixue Li, Hui Sun

**Affiliations:** Shandong Key Lab of Complex Medical Intelligence and Aging, The Second School of Clinical Medicine, Research and Translational Center for Immunological Disorders, Shandong Medical and Pharmaceutical University, Yantai, China

**Keywords:** biased signaling, G protein-coupled receptors, gut microbiota, microbiota-derived peptides, microbiota-derived proteins, microbiota-derived small molecules, β-arrestin

## Abstract

Gut microbiota modulate host metabolism, immunity, barrier integrity, and neural function through diverse classes of molecules, including small molecules such as short-chain fatty acids (SCFAs), bile acids, tryptophan metabolites, and microbe-derived peptides and proteins. G protein-coupled receptors (GPCRs) constitute a major receptor system through which the host senses microbiota-derived signals. However, current research has largely focused on ligand recognition, G protein pathways and functional phenotypes, leaving an insufficient understanding of how signals are reorganized after receptor activation to generate functional selectivity. In addition to mediating GPCR desensitization and endocytosis, β-arrestin regulates receptor trafficking, sustains endosomal signaling, and orchestrates signaling scaffold assembly, thereby providing a key mechanistic framework for understanding the spatiotemporal reprogramming of microbiota-derived signaling. This review summarizes both canonical and non-canonical GPCR signaling mechanisms, delineates the roles of β-arrestin in regulating receptor fate, biased signaling, and functional outputs. By integrating the PRESTO-Tango system and the PubMed literature, we analyzed the microbiome-related evidence for the near-complete non-olfactory GPCRome (314 conventional GPCRs) in order to assess the association between distinct classes of microbiota-derived molecules and GPCR-β-arrestin signaling. Current evidence indicates that existing studies are dominated by small-molecule signals, whereas evidence for peptides and proteins remains relatively limited. Moreover, most functional studies cannot be directly interpreted as evidence for β-arrestin-dependent mechanisms. A β-arrestin-centered conceptual framework for GPCR signaling regulation may provide a basis forunderstanding how gut microbiota-associated molecules influence host physiological functions through thespatiotemporal modulation of receptor signaling.

## 1 Introduction

The gut microbiota, one of the most complex microbial ecosystems in the human body, plays important roles in maintaining host metabolic homeostasis, immune balance, and neural regulation (Nicholson et al., 2012; Agus et al., 2021). The gut microbiota can influence host physiological processes by producing or transforming a diverse range of bioactive molecules, including small molecules such as short-chain fatty acids, bile acids, and tryptophan metabolites, as well as microbiota-derived peptides and proteins (Agus et al., 2021; Shah et al., 2025). These molecules constitute an important basis for chemical communication between the host and microbiota; however, how they are recognized at the host cellular level and translated into specific signaling outputs remains to be integrated into a unified mechanistic framework.

G protein-coupled receptors (GPCRs) are an important receptor system through which the host senses and transduces extracellular signals, participating in diverse physiological processes, including metabolism, immunity, and neural regulation, and representing one of the most important target families in drug discovery (Husted et al., 2017; Lorente et al., 2025). Previous studies have shown that a variety of microbiota-derived metabolites can act on host GPCRs. For example, short-chain fatty acids can act on FFAR2, FFAR3, and HCAR2, whereas bile acids can regulate metabolic and immune functions through receptors such as TGR5; microbiota-derived N-acyl amides and other metabolites can also activate multiple host GPCRs (Colosimo et al., 2019; Chen et al., 2023b). These findings suggest that GPCRs serve as an important bridge linking microbiota-derived chemical signals to host functional outputs.

However, GPCR-mediated signaling in response to microbiota-derived molecules is not a simple linear process but is profoundly shaped by multiple factors, including ligand properties, receptor conformation, signal transduction kinetics, and the cellular environment (Eichel et al., 2018; Staus et al., 2020; Wess et al., 2023). β-arrestin was initially regarded as a factor mediating GPCR desensitization, but it is now known to also participate in receptor internalization, intracellular trafficking, and the organization of signaling complexes (Eichel et al., 2018). These functions can influence the duration and spatial distribution of signaling and enable the same receptor to produce distinct functional outputs under different ligand conditions or cellular contexts. Therefore, β-arrestin provides an important mechanistic perspective for understanding the spatiotemporal regulation and functional selectivity of GPCR signaling.

Within this framework, microbiota-derived signals may be organized through multiple GPCR regulatory mechanisms, and β-arrestin may participate in generating context-dependent functional outputs in certain receptor systems. A functional system encompassing the near-complete non-olfactory GPCRome (314 conventional GPCRs) provides an important foundation for systematically characterizing the signaling output potential of different receptors; meanwhile, β-arrestin reporter systems, such as the Tango platform, can provide a standardized engineered readout of β-arrestin recruitment following receptor activation (Kroeze et al., 2015). This combination provides critical technical support for understanding, at the systems level, how microbiota-derived signals achieve functional diversification through the GPCR network.

Based on this background, this review focuses on the recognition and transduction of gut microbiota-derived signals within the GPCR system, with particular emphasis on the roles of β-arrestin in receptor fate regulation, spatiotemporal signal remodeling, and functional selectivity. By integrating the literature evidence for the near-complete non-olfactory GPCRome (314 conventional GPCRs), this review systematically examines diverse microbiota-derived signals, including small molecules, peptides, and proteins, and distinguishes among microbiota-associated functional evidence, well-defined evidence for microbiota-derived molecules, and direct mechanistic evidence involving β-arrestin, with the aim of further clarifying the mechanistic links among microbiota, GPCRs, and β-arrestin signaling.

## 2 Fundamentals of GPCR signal transduction

### 2.1 Definition and biological significance of GPCRs

G protein-coupled receptors (GPCRs) constitute the largest superfamily of membrane receptors in the human genome (Hauser et al., 2017; Santos et al., 2017; Zhang et al., 2024b). They are characterized by a canonical seven-transmembrane α-helical (7TM) architecture (Rasmussen et al., 2011b; Hilger et al., 2018). GPCRs mediate canonical signal transduction through heterotrimeric G proteins and also participate in non-canonical signaling processes, including receptor desensitization and internalization, through signaling regulatory proteins such as β-arrestin (Shukla et al., 2014; Staus et al., 2020). The human genome contains approximately 800 GPCR genes, of which approximately 700 encode functional receptor proteins, whereas the remaining genes do not encode functional proteins. Among the functional GPCRs, approximately 320 are olfactory receptors, whereas approximately 380 are non-olfactory GPCRs that participate extensively in the regulation of human physiological processes and constitute important targets for modern drug discovery (Hauser et al., 2017; Lorente et al., 2025).

GPCRs are widely expressed in the nervous, immune, endocrine, and cardiovascular systems, where they recognize signaling molecules such as neurotransmitters, hormones, and light (Hilger et al., 2018; Zhang et al., 2024b). Upon ligand binding, GPCRs undergo conformational changes that activate intracellular G proteins and initiate downstream signal transduction (Weis and Kobilka, 2018; Wootten et al., 2018), thereby playing essential roles in neurotransmission, energy metabolism, immune regulation, and the maintenance of physiological homeostasis (Zhang et al., 2024b). Dysregulation of GPCR function is closely associated with a variety of major diseases, including cancer, cardiovascular diseases, diabetes, and neurodegenerative disorders, making GPCRs important therapeutic targets for drug development (Wootten et al., 2018).

According to their structural and functional characteristics, GPCRs are classified into four major classes: class A (rhodopsin-like receptors, the largest class, accounting for approximately 80% of GPCRs and including adrenergic, dopamine, olfactory, and rhodopsin receptors), class B (secretin-like receptors, which bind peptide hormones such as the glucagon-like peptide-1 receptor), class C (metabotropic glutamate-like receptors, which commonly function as dimers, such as the GABA-B receptor), and class F (Frizzled/Smoothened-like receptors, which participate in Wnt signaling and the regulation of cellular development) (Weis and Kobilka, 2018; Zhang et al., 2024b).

GPCRs are of considerable value in drug discovery. To date, more than 130 non-olfactory GPCRs have been developed as clinical drug targets, accounting for approximately 35% of all approved drug targets and representing the largest family of therapeutic targets (Wootten et al., 2018). In recent years, drugs targeting non-olfactory GPCRs, including glucagon-like peptide-1 receptor agonists, β-adrenergic receptor antagonists, angiotensin II receptor antagonists, and chemokine receptor modulators, have been widely applied in the treatment of diabetes, obesity, cardiovascular diseases, neurological disorders, and immune-related diseases (DeWire and Violin, 2011; Zaïmia et al., 2023). Nevertheless, a large number of GPCRs, particularly orphan receptors, remain insufficiently characterized, providing substantial opportunities for future drug discovery (DeWire and Violin, 2011; Tang et al., 2012; Lorente et al., 2025; Ma et al., 2025).

### 2.2 Canonical and non-canonical GPCR signaling pathways

The canonical mechanism of GPCR signal transduction is dependent on heterotrimeric G proteins. Upon ligand binding, the receptor undergoes a conformational change that promotes the exchange of GDP for GTP on the Gα subunit. The activated Gα subunit subsequently dissociates from the Gβγ subunits, and both components regulate downstream effector molecules cooperatively (Hilger et al., 2018; Lorente et al., 2025). According to the subtype of the coupled G protein, GPCRs primarily transmit signals through Gαs, Gαi, Gαq, Gβγ, and G12/13 signaling pathways (Oldham and Hamm, 2008; Rasmussen et al., 2011a; Wootten et al., 2018). Among these pathways, Gαs activates adenylyl cyclase and increases intracellular cyclic AMP (cAMP) levels; Gαi inhibits adenylyl cyclase activity; Gαq stimulates phospholipase Cβ (PLCβ)-mediated generation of inositol 1,4,5-trisphosphate (IP₃) and diacylglycerol (DAG), thereby triggering Ca^2+^ release and protein kinase C (PKC) activation; and G12/13 primarily mediates cytoskeletal remodeling through the RhoGEF–RhoA signaling pathway (Rasmussen et al., 2011a). Collectively, these canonical signaling pathways constitute the fundamental framework through which GPCRs mediate cellular responses (Wootten et al., 2018).

However, with the deepening of GPCR research, it has become increasingly evident that GPCR function is not solely dependent on heterotrimeric G proteins (Reiter and Lefkowitz, 2006; Eichel et al., 2018). Following receptor activation, GPCRs can also recruit signaling regulatory proteins, such as β-arrestins, which participate in receptor desensitization, receptor internalization, and G protein-independent signal transduction (Reiter and Lefkowitz, 2006; Shenoy and Lefkowitz, 2011; Nuber et al., 2016; Eichel et al., 2018; Grundmann et al., 2018; Wess et al., 2023). β-arrestins not only terminate conventional G protein-mediated signaling but also function as signaling scaffold proteins that assemble and activate multiple downstream signaling pathways (Eichel et al., 2018; Staus et al., 2020), thereby forming a non-classical signaling network parallel to the G protein pathway. Accordingly, β-arrestins have gradually emerged as a key entry point for understanding GPCR functional plasticity and for developing biased drugs (Nuber et al., 2016; Peterson and Luttrell, 2017; Eichel et al., 2018; Xiao et al., 2021; Wess et al., 2023). Growing evidence indicates that distinct ligands and conditions induce different receptor conformational states, thereby selectively activating specific signaling pathways, a process referred to as “biased signaling” (Rajagopal et al., 2010; Klein Herenbrink et al., 2016; Maeda et al., 2018; Xiao et al., 2021). This concept has expanded the traditional view that GPCRs transmit signals through a single signaling mode and provides an important theoretical foundation for the development of next-generation biased drugs with improved efficacy and reduced adverse effects (Jayakody et al., 2025).

## 3 β-arrestin-mediated GPCR signaling reprogramming and research strategies

### 3.1 β-arrestin signaling

β-arrestin was initially regarded as a negative regulator of GPCR desensitization. Following receptor activation, G protein-coupled receptor kinases (GRKs) phosphorylate the receptor, allowing β-arrestin to bind to the activated receptor and prevent further coupling with G proteins, thereby terminating canonical G protein-mediated signaling. As research has progressed, the functional roles of β-arrestin have been substantially expanded. In addition to mediating receptor desensitization, β-arrestin also participates in GPCR internalization, intracellular trafficking, and the organization of downstream signaling, thereby coordinating receptor trafficking with signal transduction. These signaling processes are illustrated in Figure 1.

**Figure 1:**
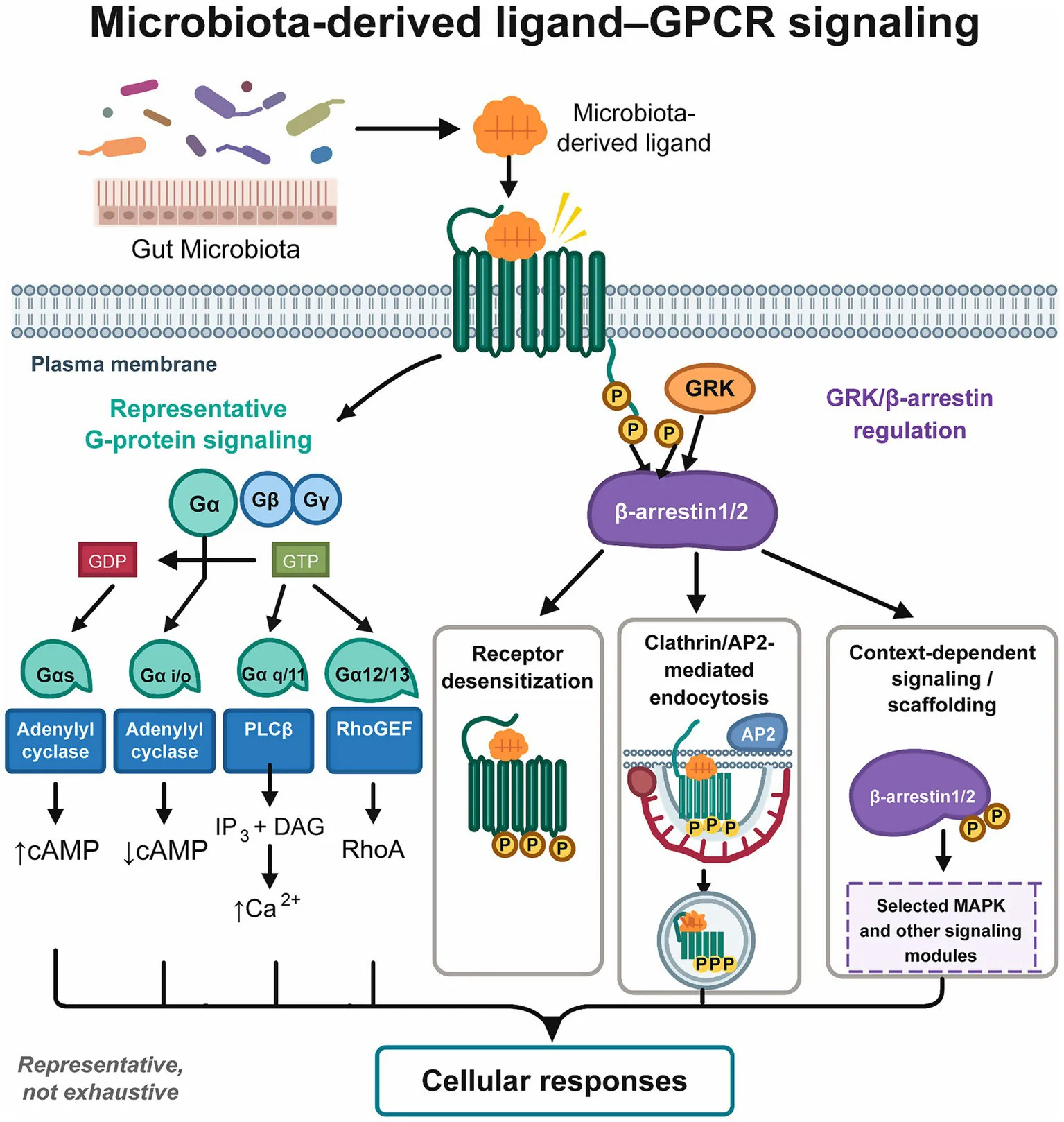
Gut microbiota-derived ligand–GPCR signaling. The figure illustrates G protein signaling and GRK/β-arrestin-mediated receptor regulation following activation of host GPCRs by gut microbiota-derived ligands.

### 3.2 β-arrestin regulates receptor fate and the spatiotemporal organization of GPCR signaling

The role of β-arrestin in reshaping receptor fate is first reflected in its regulation of GPCR internalization and intracellular trafficking. Studies have demonstrated that the phosphorylation barcode is not merely a simple “switch” but rather an information-encoding system that specifies distinct receptor fates. Following receptor activation, distinct phosphorylation barcodes induce distinct β-arrestin conformational states, thereby influencing the stability of receptor–β-arrestin complexes and the efficiency of receptor internalization (Bous et al., 2022; Sun et al., 2025).

Recent cryo-EM studies of PTH1R-β-arrestin1 and KOR-β-arrestin1 complexes have further clarified the structural basis of β-arrestin engagement with GPCRs. Within a broadly conserved core-engagement framework, the orientation of β-arrestin and its local receptor contacts can vary across different GPCRs and even between conformational states of the same receptor. These studies further highlight the conformational plasticity of GPCR-arrestin coupling and show that a shared core engagement mode can accommodate receptor- and state-specific structural variations (Han et al., 2026; Zhai et al., 2026; Zhao et al., 2026).

Therefore, receptor trafficking not only determines the intracellular destination of receptors but also directly influences the duration of GPCR signaling and the magnitude of cellular responses (Chen et al., 2025). In the case of VIPR1, β-arrestin participates in plasma-membrane signal desensitization, whereas receptor internalization and endosomal cAMP signaling can occur independently of β-arrestin, indicating mechanistic uncoupling among these processes (Blythe and von Zastrow, 2024). Accordingly, receptor trafficking itself has become an integral component of GPCR signal regulation rather than a passive process that occurs after termination of canonical signaling.

In the past, GPCR signaling was generally considered to occur primarily at the plasma membrane, and receptor internalization was regarded as synonymous with signal termination. However, accumulating evidence indicates that the relationships among plasma-membrane desensitization, receptor internalization, and sustained endosomal signaling are receptor-dependent. β-arrestin may participate in some of these processes, whereas internalization and endosomal signaling can be retained in its absence for certain receptors. Through live-cell imaging and structural studies, it has been shown that β-arrestin can adopt a “receptor-ready” membrane-associated state before receptor engagement and, under the regulation of membrane lipids such as phosphatidylinositol 4,5-bisphosphate (PIP₂), promotes the assembly and stabilization of receptor complexes, thereby influencing complex residence time and signaling duration (Janetzko et al., 2022; Gomes et al., 2025). Therefore, the local membrane microenvironment also represents an important regulatory factor that determines β-arrestin-mediated signaling output and provides a mechanistic explanation for the distinct functional effects elicited by the same receptor in different cellular contexts.

In addition, β-arrestin serves as an important signaling scaffold (Yang et al., 2018). Different receptor conformations and phosphorylation patterns can induce distinct activation states of β-arrestin, thereby enabling the selective assembly of downstream signaling modules, including ERK, JNK, and p38 MAPK, and giving rise to signaling outputs with spatial restriction and functional selectivity. This scaffolding function further links receptor activation to biological processes such as inflammation, metabolism, and tissue remodeling. Notably, β-arrestin not only participates in the regulation of receptor fate but also directly influences disease-associated phenotypes, further highlighting its pivotal role as an organizer of GPCR signaling networks.

Taken together, β-arrestin establishes a continuous regulatory axis from receptor activation to phenotypic output by coordinating receptor trafficking, the spatiotemporal redistribution of signaling, and scaffold-mediated signal integration. Ligand-dependent differences in β-arrestin recruitment and receptor–β-arrestin complex dynamics may influence receptor trafficking and signal organization, thereby providing a mechanistic basis for differential signaling outputs; whether these differences constitute β-arrestin bias requires quantitative comparison with G protein signaling.

### 3.3 β-arrestin-biased signaling

Biased signaling refers to the phenomenon whereby different ligands selectively drive downstream signaling pathways, such as those mediated by G proteins or β-arrestin, by stabilizing distinct active conformations of the same GPCR, thereby producing different functional outputs (Jayakody et al., 2025). This concept extends the traditional receptor pharmacology framework centered on agonist efficacy by demonstrating that GPCRs are not simply “on/off” switches but dynamic regulatory systems capable of adopting multiple active conformational states and encoding distinct signaling modes.

Existing studies have demonstrated that the generation of biased signaling arises from multilevel conformational regulation following receptor activation. Different ligands initially induce distinct receptor conformations, which subsequently influence the mode of GRK recruitment and the establishment of phosphorylation barcodes. Distinct phosphorylation barcodes, in turn, determine the binding conformation and functional state of β-arrestin, ultimately governing receptor trafficking and downstream signaling outputs (Cheng et al., 2024; Kumari et al., 2024). Meanwhile, the membrane lipid environment, including PIP₂, together with the intrinsic conformational plasticity of β-arrestin, further regulates the assembly, stability, and kinetic properties of receptor–β-arrestin complexes, thereby conferring a marked dependence of biased signaling on the cellular context.

Therefore, biased signaling is not a single molecular event but rather the integrated outcome of ligand binding, receptor conformation, GRK-mediated encoding, the membrane microenvironment, and β-arrestin conformation. Interestingly, this continuous regulatory process not only explains the molecular basis by which the same receptor produces diverse biological effects but also provides a theoretical framework for the subsequent design of biased ligands and the precise regulation of GPCR signaling.

### 3.4 Research strategies and translational value of β-arrestin signaling

#### 3.4.1 Translational strategies for biased pharmacology

The concept of β-arrestin-biased signaling has driven a paradigm shift in GPCR drug discovery from receptor activation toward signaling modulation. Current strategies primarily include structure-guided design of biased ligands (Wang et al., 2024), allosteric modulation (Ippolito et al., 2023), regulation of GRK-mediated phosphorylation barcodes (Sánchez-Soto et al., 2023; Matthees et al., 2024), and intervention in the membrane microenvironment (Janetzko et al., 2022), all of which aim to optimize therapeutic efficacy while minimizing adverse effects through the redistribution of signaling outputs.

The μ-opioid receptor (μOR) (Stahl et al., 2021), angiotensin II type 1 receptor (AT1R) (Boerrigter et al., 2011; Reiter et al., 2012; Kawakami et al., 2022), and glucagon-like peptide-1 receptor (GLP-1R) (Zheng et al., 2024) represent three representative models of biased pharmacology. These receptors achieve functional selectivity in different disease settings by enhancing G protein signaling, preserving protective β-arrestin signaling, or optimizing receptor trafficking and signaling persistence, respectively. Nevertheless, although biased signaling provides an important theoretical basis for the precise regulation of GPCR-targeted therapeutics, its clinical translation still requires further validation.

#### 3.4.2 The tango reporter system

The Tango reporter system detects ligand-induced GPCR-β-arrestin interactions through transcriptional reporter signals. Following receptor activation, the β-arrestin2-TEV protease fusion protein is recruited to the receptor, facilitating cleavage at the TEV recognition site linked to the receptor and releasing the transcription factor tTA, which subsequently drives reporter gene expression (Barnea et al., 2008). Tango converts receptor-β-arrestin interactions into amplified transcriptional outputs, providing a sensitive screening approach without requiring prior knowledge of the receptor’s G-protein coupling profile. The underlying principle is illustrated in Figure 2.

**Figure 2:**
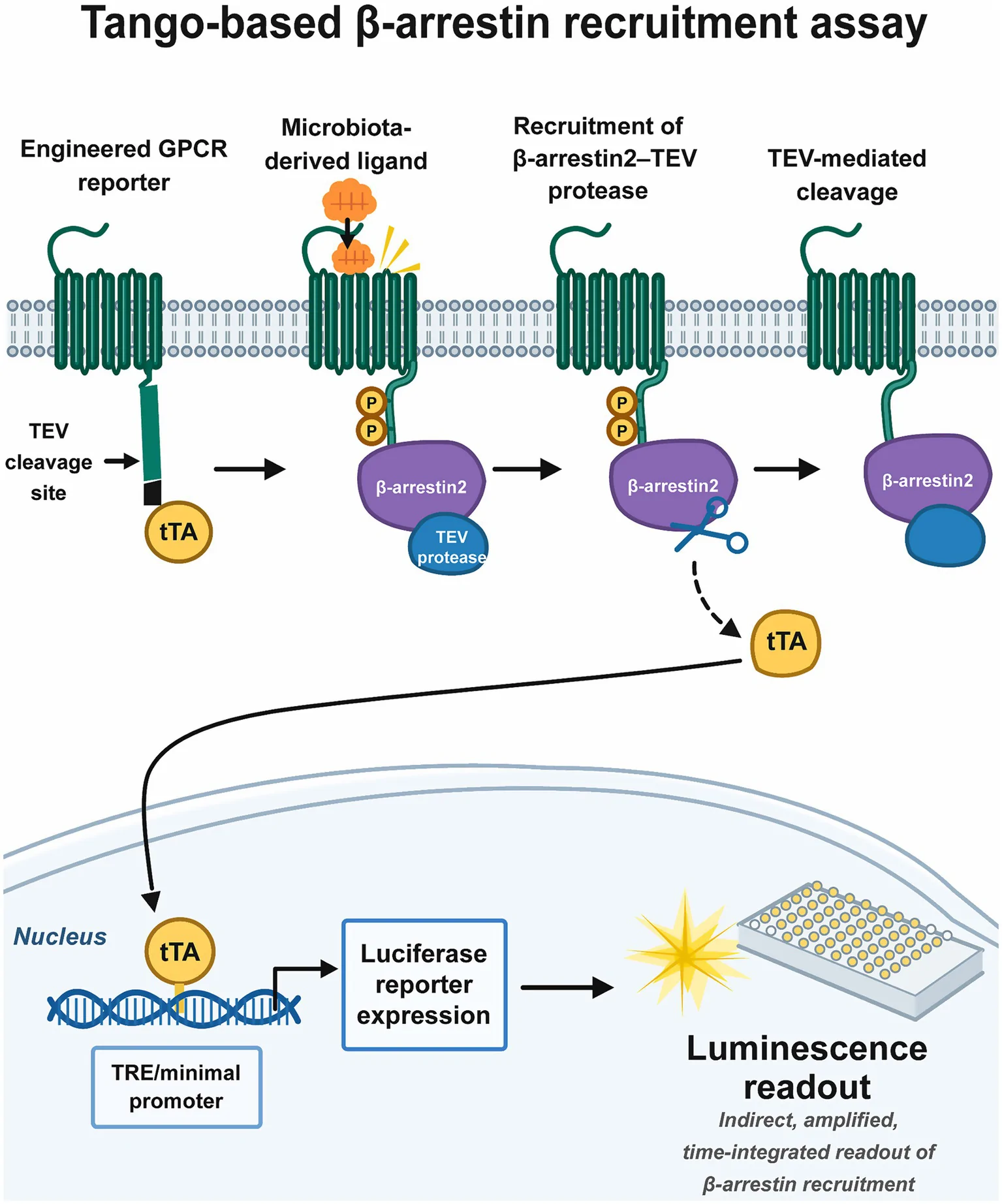
β-Arrestin recruitment detection by the Tango reporter system. The Tango system converts receptor activation into a detectable transcriptional reporter signal through β-arrestin recruitment and TEV-mediated cleavage.

Building on this approach, PRESTO-Tango extended β-arrestin-based screening to a broad range of non-olfactory GPCRs, enabling ligand screening at the GPCRome scale and facilitating the identification of candidate ligands for understudied and orphan receptors (Kroeze et al., 2015). By adopting β-arrestin recruitment as a unified functional endpoint, this platform enhances the efficiency of large-scale screening. However, it also necessitates that screening signals be interpreted in the context of the characteristics and limitations of the assay itself.

In the Tango assay, the reporter signal reflects the combined effects of multiple steps, including β-arrestin recruitment, TEV-mediated cleavage, transcription factor release, and subsequent reporter gene expression, rather than providing a direct, real-time measurement of receptor-β-arrestin binding. Thus, although Tango is well suited for detecting ligand-induced recruitment events, it has limitations in resolving recruitment kinetics. Meanwhile, receptor overexpression, reporter-component fusion, and experimental conditions, including ligand concentration, may also influence the observed receptor signaling responses or trafficking behavior (Spillmann et al., 2020). Therefore, Tango signals should be interpreted in light of the assay’s underlying detection mechanism and experimental conditions to determine the specific biological processes they reflect.

Orthogonal recruitment assays, such as BRET/NanoBRET and PathHunter, can provide complementary evidence through distinct detection principles. The β-arrestin dependence can be validated through causal perturbation of ARRB1 and/or ARRB2, whereas β-arrestin bias can be assessed by quantitatively comparing β-arrestin and the corresponding G-protein pathway responses under comparable experimental conditions (Zhang and Xie, 2012; Luttrell et al., 2018; Kolb et al., 2022). Overall, Tango provides an effective approach for β-arrestin recruitment screening; however, conclusions regarding β-arrestin dependence and signaling bias require further validation using orthogonal assays, causal perturbations, and pathway-level comparisons.

## 4 Gut microbiota-derived molecules and GPCR–β-arrestin signaling

### 4.1 Current research on gut microbiota-derived signals and GPCRs

Gut microbiota-derived small molecules, peptides, and proteins represent important sources of signals through which the host senses changes in the intestinal microenvironment. To further define the associations between these microbiota-derived signals and host GPCRs and β-arrestins, we conducted a structured literature survey and synthesized the relevant evidence.

The literature search adopted a stepwise narrowing strategy. Using the non-olfactory human GPCRs covered by PRESTO-Tango as the receptor framework, we first searched the PubMed database for relevant studies published over the past 15 years. We then further screened these studies for those involving the gut microbiota and subsequently identified publications containing explicit evidence for microbiota-derived molecules or microbiota-associated molecular signals, which were categorized into metabolic small molecules, peptides, and proteins. Subsequently, through manual screening of titles and abstracts and, when necessary, full-text examination, we further identified studies addressing specific “microbiota-derived molecule–GPCR” interactions and β-arrestin-related mechanisms. The detailed search workflow and the inclusion and exclusion criteria applied at each stage are shown in Figure 3. Using the near-complete non-olfactory GPCRome (314 conventional GPCRs) as the initial receptor set, we identified 137 receptors associated with the gut microbiota, of which 56 had explicit evidence for microbiota-derived molecular signals. The overall research intensity across receptors does not correspond closely to the distribution of gut microbiota-related evidence (Figure 4). Microbiota-related evidence and well-defined evidence for specific microbiota-derived molecules are relatively concentrated for FFAR2/GPR43, FFAR3/GPR41, GPBAR1/TGR5, and HCAR2/GPR109A, suggesting that current research has focused primarily on metabolite-sensing GPCRs; meanwhile, evidence for microbiota-derived molecules remains limited for some highly studied receptors.

**Figure 3:**
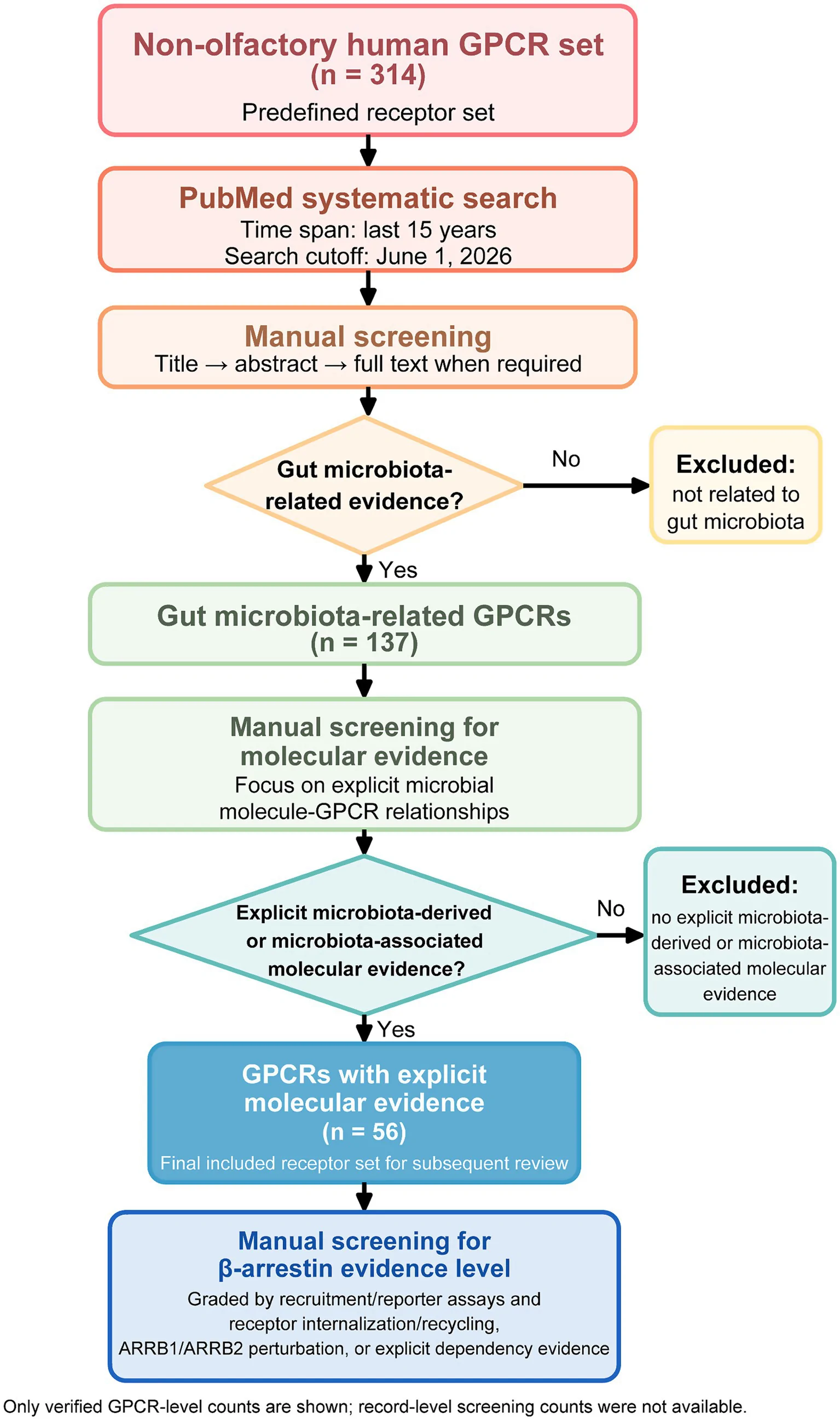
Screening of gut microbiota-related GPCRs and β-arrestin evidence. The figure shows the stepwise screening of GPCRs for gut microbiota-related evidence, explicit microbiota-derived or microbiota-associated molecular evidence, and β-arrestin-related evidence.

**Figure 4:**
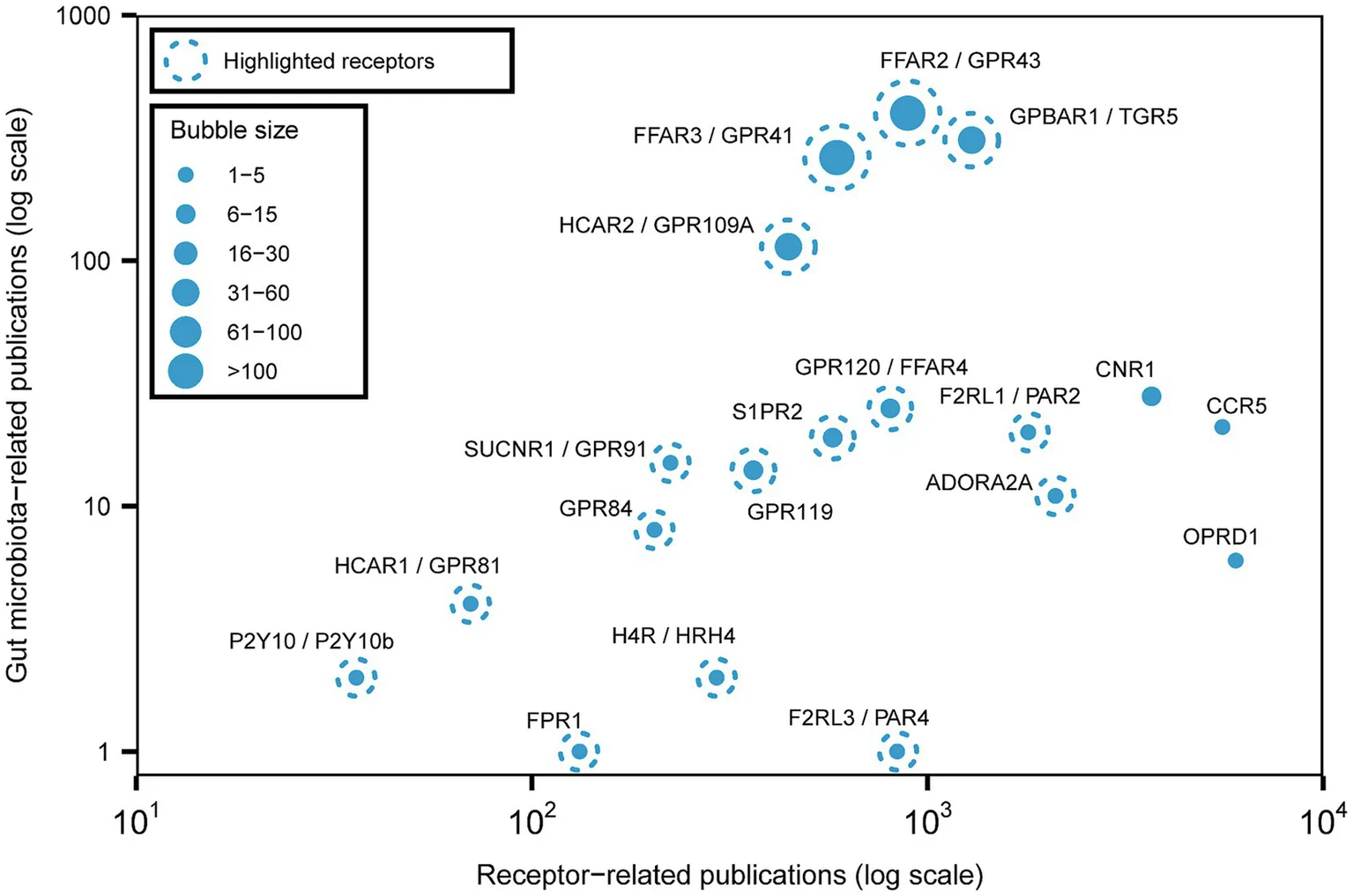
Bubble plot showing the distribution of evidence for selected GPCRs. The x-axis represents the number of receptor-related publications, and the y-axis represents the number of gut microbiota-related publications, both on a logarithmic scale. Bubble size represents the total amount of evidence associated with microbiota-derived small molecules, peptides, and proteins for each receptor, while the dashed outer circles indicate the receptors discussed in detail in this review.

The composition of the evidence for different types of microbiota-derived molecules is markedly skewed (Figure 5). A total of 449 publications provided evidence for small molecules, whereas only 4 publications provided evidence for proteins involving F2RL1/PAR2 and F2RL3/PAR4, and 1 publication provided evidence for peptides involving FPR1. These findings indicate that GPCR-related research remains strongly concentrated on small molecules, whereas mechanisms involving microbiota-derived proteins and peptides require further investigation.

**Figure 5:**
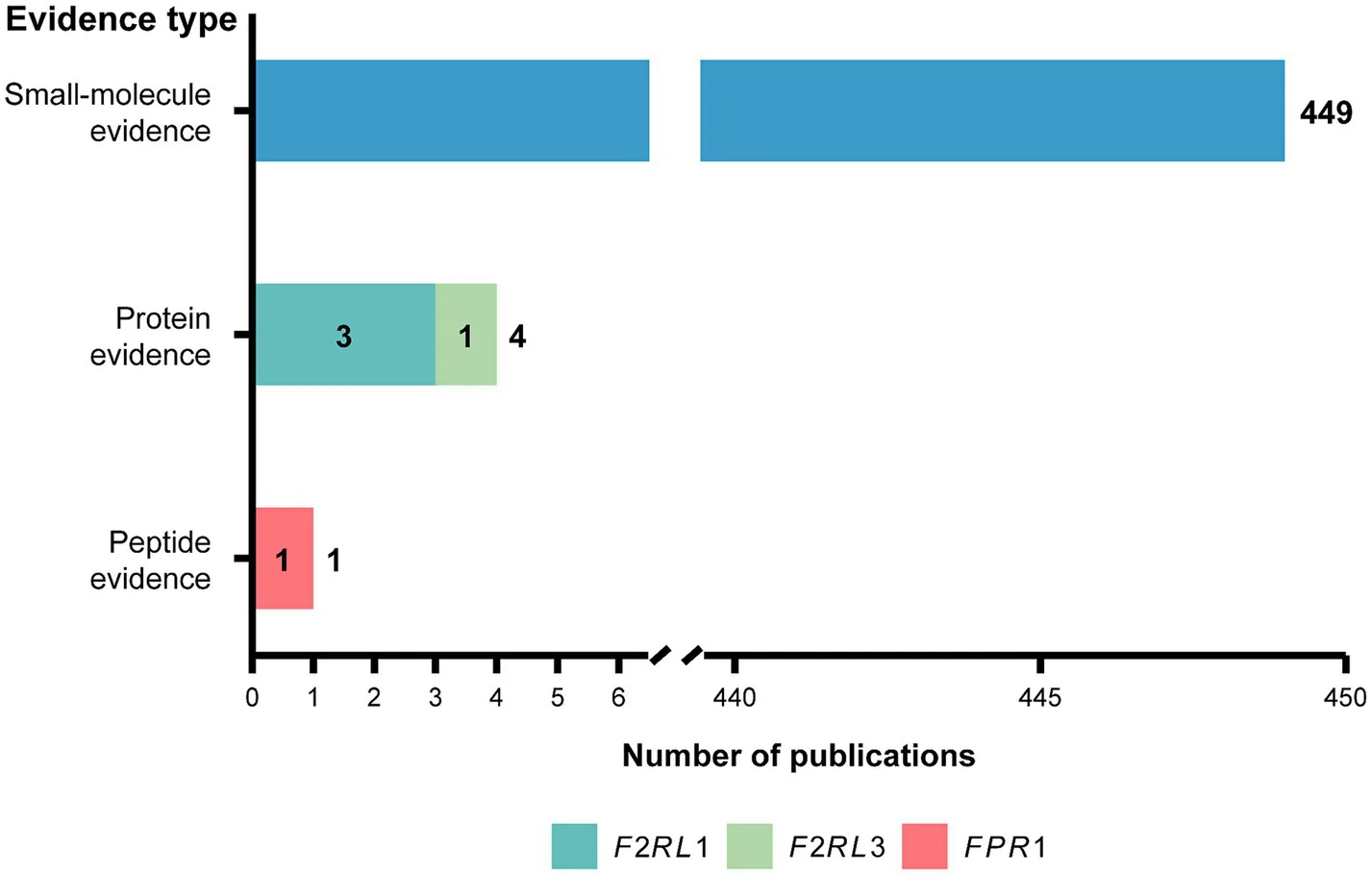
Distribution of microbiota-derived molecular evidence associated with GPCRs. The bar chart shows the numbers of publications linking gut microbiota-derived small molecules, proteins, and peptides to GPCRs, with colored segments indicating the corresponding receptors.

Marked differences were also observed across evidence levels for the selected GPCRs (Figure 6). Some receptors have relatively abundant evidence for microbiota-derived molecules particularly small molecules, yet the evidence supporting β-arrestin mechanisms remains limited, with higher-level evidence concentrated in only a small number of receptors. These findings indicate a marked imbalance between the current evidence for microbiota-derived molecules and the mechanistic characterization of β-arrestin.

**Figure 6:**
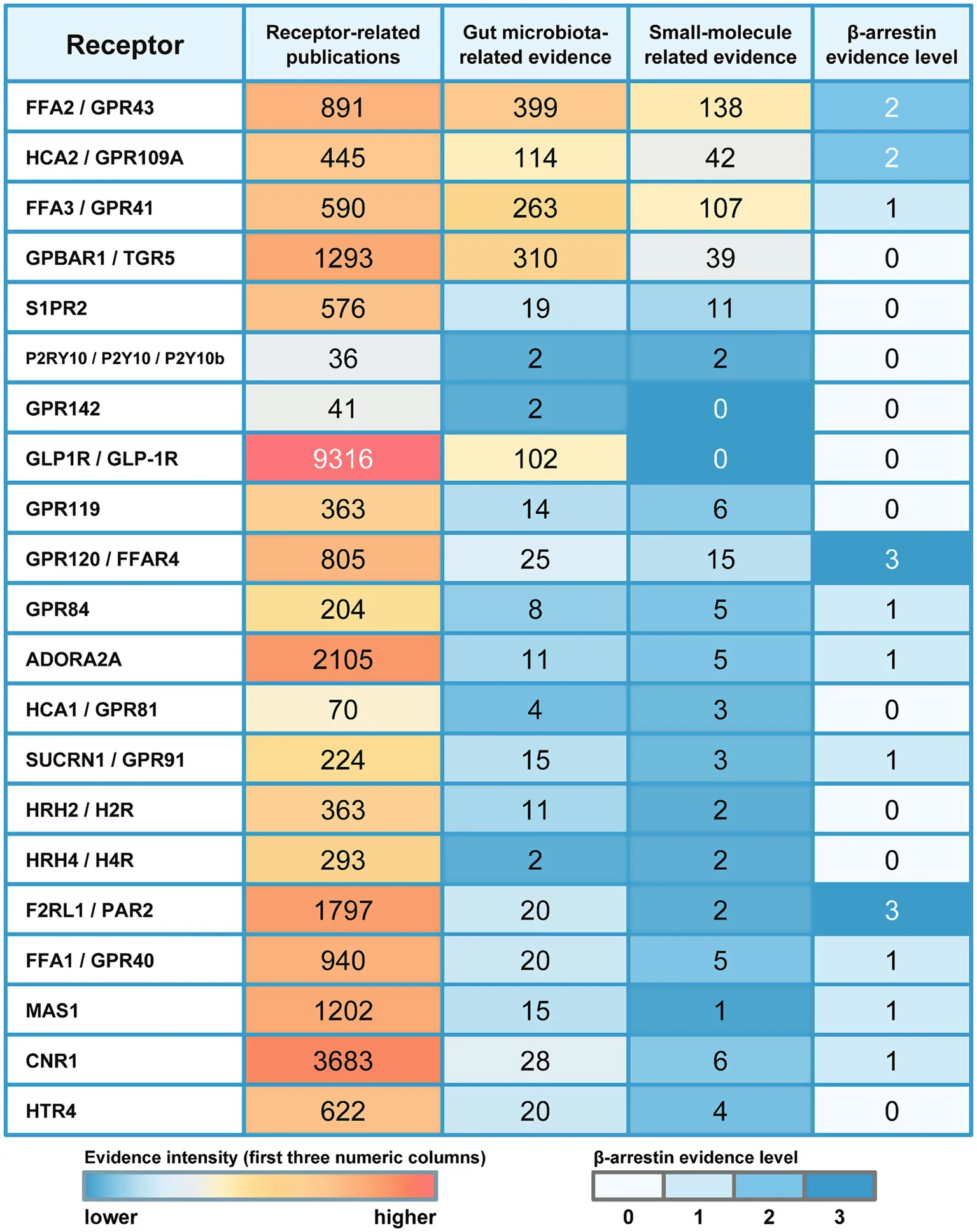
Evidence distribution and levels of β-arrestin mechanistic evidence for selected GPCRs. The first three columns indicate the numbers of receptor-related, gut microbiota-derived, and small-molecule-related publications, respectively, with darker blue indicating higher numbers; the final column indicates the level of β-arrestin mechanistic evidence.

Based on the evidence landscape described above, this section will successively review GPCR-β-arrestin signaling mediated by short-chain fatty acids, bile acids, indole-related and other microbiota-derived small molecules, as well as microbiota-derived peptides and proteins, with particular emphasis on mechanistic evidence for β-arrestin recruitment, receptor internalization, ARRB1/ARRB2 dependence, and biased signaling.

### 4.2 GPCR signaling and β-arrestin regulation by gut microbiota-derived small molecules

#### 4.2.1 Short-chain fatty acids

Short-chain fatty acids (SCFAs), mainly acetate, propionate, and butyrate, are key metabolites produced by gut microbiota through the fermentation of dietary fiber. In addition to serving as energy substrates for colonic epithelial and other cells, they play critical roles in host metabolism, immune regulation, and inflammation control (van der Hee and Wells, 2021; Mukhopadhya and Louis, 2025). With their high concentration in the intestinal lumen, SCFAs can also enter local tissues or systemic circulation to function as metabolic and immune signaling molecules, effectively linking the “diet–microbial metabolism state” with host metabolic and immune regulation (Mann et al., 2024).

The receptor recognition of SCFAs primarily relies on metabolite-sensing GPCRs, with FFAR2/GPR43, FFAR3/GPR41, and GPR109A/HCAR2 forming the core receptors (Macia et al., 2015; Sivaprakasam et al., 2016; Kimura et al., 2020; van der Hee and Wells, 2021).

Existing evidence suggests that the functions of the SCFA-GPCR pathway extend from the maintenance of intestinal homeostasis to broader regulatory roles in metabolism, immune function, and extraintestinal organs (van der Hee and Wells, 2021; Lymperopoulos et al., 2022; Mann et al., 2024; Mukhopadhya and Louis, 2025; Xu et al., 2025a). Locally in the gut, FFAR2/GPR43 and GPR109A/HCAR2 mediate the protective effects of high-fiber diets or SCFAs on colonic inflammation and epithelial homeostasis (Singh et al., 2014; Macia et al., 2015; Sivaprakasam et al., 2016). In immune regulation, GPR109A/HCAR2 mediates the anti-inflammatory and antitumor effects induced by butyrate, whereas FFAR2/GPR43 and FFAR3/GPR41 suppress inflammatory activation of T cells and myeloid cells (Singh et al., 2014; Porbahaie et al., 2023; Mann et al., 2024; Yu et al., 2024). At the metabolic and neuroendocrine levels, FFAR2/GPR43 regulates insulin signaling in adipose tissue, while FFAR3/GPR41 is involved in the regulation of sympathetic nerve activity and energy expenditure (Kimura et al., 2011, 2013). Both receptors also contribute to the release of GLP-1 and PYY from enteroendocrine cells (Cani et al., 2013; Kimura et al., 2020). Further studies on extraintestinal organs reveal that FFAR2/GPR43 influences atrial fibrillation-associated inflammation and atrial remodeling through the GPR43-NLRP3 axis. Additionally, FFAR2 contributes to gallbladder mucus secretion and contraction via the FFAR2-tuft cell axis and regulates follicular maturation through the GPR43-gWAT-leptin axis (Keshavarz et al., 2022; Zuo et al., 2022; Xu et al., 2025a).

In studies on SCFA receptors, G protein signaling remains the most well-established mechanistic framework, while β-arrestin offers a complementary perspective for understanding the persistence, spatial localization, and trafficking regulation of receptor signaling (Shenoy and Lefkowitz, 2011; Macia et al., 2015; Sivaprakasam et al., 2016). As an analogous example, pharmacological niacin activates GPR109A and promotes β-arrestin1-dependent cPLA2 activation and arachidonic acid release, contributing to cutaneous flushing in mice (Walters et al., 2009). Whether microbiota-derived SCFAs engage β-arrestin-mediated mechanisms at GPR109A, FFAR2/GPR43, or FFAR3/GPR41 remains unclear (Shenoy and Lefkowitz, 2011; Thomsen et al., 2016). This example provides a rationale for investigating β-arrestin involvement in the signaling and trafficking of SCFA-related GPCRs.

Based on this body of evidence, SCFAs are not only crucial metabolites derived from the gut microbiota but also key signaling molecules in host homeostasis regulation. At present, mechanistic studies on SCFA-related receptors primarily focus on G protein signaling and functional phenotypes. Their effects span regulation of the intestinal barrier, inflammation control, immunometabolism, enteroendocrine function, and some extraintestinal organ functions. Building upon this foundation, research on β-arrestin can further uncover novel mechanisms underlying the regulatory roles of SCFA receptors in trafficking control, signal persistence, and tissue-specific functions.

#### 4.2.2 Bile acid-derived signaling molecules

Bile acids (BAs) are signaling molecules generated from hepatic cholesterol metabolism and further transformed by the gut microbiota within the intestinal lumen (Wahlström et al., 2016; Perino et al., 2021; Collins et al., 2023; Larabi et al., 2023). Primary bile acids are synthesized in the liver, conjugated with glycine or taurine, and subsequently secreted into the intestine via bile (Wahlström et al., 2016; Perino et al., 2021; Collins et al., 2023; Larabi et al., 2023). Within the intestinal lumen, the gut microbiota remodel the bile acid pool through deconjugation, dehydroxylation, oxidation, and epimerization, thereby generating secondary bile acids and a diverse array of modified bile acid species (Wahlström et al., 2016; Perino et al., 2021; Collins et al., 2023; Larabi et al., 2023). Consequently, the composition of the bile acid pool is jointly shaped by host metabolism, enterohepatic circulation, dietary composition, and the structure of the gut microbial community (Wahlström et al., 2016; Perino et al., 2021; Collins et al., 2023; Larabi et al., 2023).

Among bile acid receptors, the nuclear receptor farnesoid X receptor (FXR) and the G protein-coupled receptor Takeda G protein-coupled receptor 5 (TGR5/GPBAR1) represent the best-characterized signaling systems (Pols et al., 2011; Wahlström et al., 2016; van Nierop et al., 2017; Perino et al., 2021). FXR belongs to the nuclear receptor superfamily and primarily regulates bile acid synthesis, transport, and metabolic homeostasis (Wahlström et al., 2016; Perino et al., 2021). TGR5 is a membrane receptor that has been well studied in bile acid-related GPCRs and is commonly associated with Gs-cAMP signaling, participating in the regulation of enteroendocrine function, metabolism, and inflammation (Pols et al., 2011; van Nierop et al., 2017; Perino et al., 2021). In addition to TGR5, other GPCRs not primarily classified as bile acid receptors, including S1PR2 and P2Y10, can also respond to specific bile acids or conjugated bile acids, further expanding the bile acid-responsive GPCR network in a ligand- and context-dependent manner (Shibuya et al., 2021; Miao et al., 2022; Xu et al., 2025b).

Bile acid–GPCR signaling participates in the regulation of enteroendocrine function, metabolic homeostasis, epithelial barrier integrity, and immune inflammatory responses (Pols et al., 2011; Wahlström et al., 2016; van Nierop et al., 2017; Perino et al., 2021; Collins et al., 2023; Larabi et al., 2023). The gut microbiota can alter the bile acid composition in the ileum and influence postprandial GLP-1 secretion through TGR5 (Wang et al., 2023). Restoration of the gut microbiota or supplementation with relevant bile acids can re-establish GLP-1 responsiveness, suggesting that TGR5-mediated bile acid signaling participates in microbiota-dependent regulation of enteroendocrine function (Wang et al., 2023). TGR5 activation is also associated with energy expenditure, glucose homeostasis, colonic epithelial integrity, and the improvement of metabolic kidney injury (Pols et al., 2011; Wang et al., 2016; van Nierop et al., 2017; Zhai et al., 2021; Chen et al., 2023a). In immune regulation, DCA can partially upregulate the expression of ICAM-1 and VCAM-1 in intestinal vascular endothelial cells through S1PR2, thereby promoting lymphocyte adhesion and migration, whereas TDCA can activate Ca^2+^ and RhoA signaling through P2Y10 and maintain the residence of intestinal innate lymphoid cells (Shibuya et al., 2021; Xu et al., 2025b).

β-arrestin further expands the mechanism content of the TGR5 signaling (Cahill et al., 2017; Pavlos and Friedman, 2017; Hu et al., 2019; Perino et al., 2021). In studies related to TGR5, viral infection has been shown to induce intracellular bile acid accumulation and promote the recruitment of SRC by TGR5 through β-arrestin, thereby forming a signaling axis (Hu et al., 2019). This TGR5–β-arrestin–SRC axis enhances IRF3 phosphorylation and type I interferon responses, thereby promoting innate antiviral immunity (Hu et al., 2019). This mechanism suggests that TGR5 can function not only through the canonical Gs–cAMP pathway but also participate in immune defense through β-arrestin-dependent scaffold signaling in specific cellular contexts (Hu et al., 2019). In contrast, studies on DCA–S1PR2 signaling, TDCA–P2Y10 signaling, and TGR5-mediated regulation of GLP-1 secretion mainly support functional effects mediated by bile acid–GPCR signaling, whereas their dependence on β-arrestin still requires dedicated experimental validation (Shibuya et al., 2021; Wang et al., 2023; Xu et al., 2025b).

In summary, bile acid signaling is jointly shaped by host bile acid metabolism and microbial transformation and participates in the regulation of enteroendocrine function, metabolic homeostasis, barrier maintenance, and immune cell localization through GPCRs, including TGR5, S1PR2, and P2Y10. Among these receptors, TGR5 is currently the bile acid-responsive GPCR supported by the most extensive evidence. It mediates GLP-1 secretion and metabolic regulation through the canonical Gs-cAMP pathway and can also promote innate antiviral immunity through β-arrestin-SRC signaling under specific conditions, such as viral infection. Therefore, β-arrestin should not be generalized as a universal mechanism underlying bile acid-GPCR signaling. Its established involvement in TGR5 signaling suggests that future studies of bile acid receptors should further investigate receptor internalization, signaling scaffolds, and biased signaling outputs beyond G protein-mediated pathways.

#### 4.2.3 Indole-derived small molecules

Indole derivatives are primarily produced by the metabolism of tryptophan by the gut microbiota and serve as important small-molecule signals linking dietary intake, gut microbial composition, and host mucosal homeostasis (Zhang et al., 2021; Ye et al., 2022; Xue et al., 2023). Representative metabolites include indole, indole-3-propionic acid, and indole-3-lactic acid (Zhang et al., 2021; Ye et al., 2022). Some microbiota-derived indole metabolites have been reported to act on host GPCRs, providing a basis for further investigation of their links to β-arrestin signaling.

Among GPCRs, HCA3 (GPR109B) provides a relatively direct link between microbiota-derived indole-3-lactic acid and β-arrestin signaling. Bifidobacterium-derived indole-3-lactic acid can activate HCA3 and suppress IL-12p70 production in human monocytes through an HCA3-dependent mechanism (Laursen et al., 2021). In HCA3-expressing HEK293-T cells, the PathHunter β-arrestin assay further showed that indole-3-lactic acid induced β-arrestin-2 recruitment, providing direct receptor-proximal evidence that a microbiota-derived indole metabolite can activate a GPCR and engage β-arrestin-related signaling (Peters et al., 2020). However, whether β-arrestin directly mediates HCA3-associated physiological effects remains to be established.

GPR35, by contrast, provides an informative mechanistic reference. Its ligand kynurenic acid (KYNA) is primarily associated with the host kynurenine pathway and is therefore not a strictly microbiota-derived ligand, although its production can be modulated by the gut microbiota (Miyamoto et al., 2023; Xue et al., 2023). Using a synthetic small-intestinal microbial community and metabolite measurements, Jansma et al. (2023) linked microbiota-mediated changes in kynurenine metabolism to KYNA production and further discussed its potential intestinal relevance in light of the known activity of KYNA at GPR35. Together with previous evidence that KYNA can induce β-arrestin-2 recruitment to GPR35 (Jenkins et al., 2011), these findings allow microbiota-associated regulation of KYNA production and GPR35–β-arrestin signaling to be considered within a common mechanistic framework. Current evidence suggests potential continuity among these steps, but a complete microbiota–KYNA–GPR35–β-arrestin signaling axis has yet to be experimentally established. Accordingly, this axis is better regarded as a mechanistic reference for linking microbiota-regulated metabolic signaling to GPR35–β-arrestin mechanisms rather than as direct evidence of signaling by a microbiota-derived ligand.

Overall, the indole-3-lactic acid–HCA3 axis provides relatively direct receptor-proximal evidence of GPCR activation and β-arrestin-2 recruitment by a microbiota-derived tryptophan metabolite, whereas the KYNA–GPR35 axis mainly serves as a mechanistic reference involving a microbiota-regulated host metabolite. Whether β-arrestin directly mediates the physiological effects associated with either pathway remains to be determined.

#### 4.2.4 Other small molecules

Other small molecules provide important additions to microbiota-derived metabolite-GPCR signaling. Based on GPCR functional screening of metabolites derived from human gut bacteria and multi-omics mechanistic studies, biogenic amines, purine metabolites, lipid mediators, organic acids, and sphingolipids have also been shown to participate in the regulation of host GPCRs (Lee et al., 2018; Chen et al., 2019; Otake-Kasamoto et al., 2022; Igarashi et al., 2023; Dong et al., 2025).

Lipid metabolites currently represent the subgroup supported by the most extensive mechanistic evidence. LysoPS, LPC, OEA, 12-methylmyristic acid, and decanoic acid are linked to receptors including P2Y10/P2Y10b, GPR119, GPR120/FFAR4, and GPR84, respectively (Otake-Kasamoto et al., 2022; Igarashi et al., 2023; Xu et al., 2023; Zhao et al., 2023; Zha et al., 2025). These receptors participate in the regulation of intestinal inflammation, neurodegeneration, feeding behavior, lipid metabolism, and skeletal muscle function (Otake-Kasamoto et al., 2022; Igarashi et al., 2023; Xu et al., 2023; Zhao et al., 2023; Zha et al., 2025). Among them, Crohn’s disease-associated microbiota-derived LysoPS activates Gα12/13-Rho-ROCK signaling through P2Y10/P2Y10b, thereby promoting glycolysis, chromatin accessibility, and IFN-γ production in Th1 cells and consequently exacerbating intestinal inflammation (Otake-Kasamoto et al., 2022). LPC suppresses ACSL4-associated ferroptosis through GPR119 and attenuates Alzheimer’s disease-like pathology, whereas OEA regulates satiety and feeding behavior during the refeeding phase through GPR119 (Igarashi et al., 2023; Zha et al., 2025). Activation of GPR120/FFAR4 by 12-methylmyristic acid improves lipid metabolism and glucose homeostasis, whereas decanoic acid promotes myoblast differentiation and muscle hypertrophy through GPR84-PI3K/AKT signaling (Xu et al., 2023; Zhao et al., 2023).

Purine metabolites and organic acids further connect immunometabolism, barrier repair, and distal organ function. Inosine, lactate, and succinate participate in obesity-associated inflammation, intestinal epithelial regeneration, intestinal barrier dysfunction, and myocardial ischemia–reperfusion injury through the adenosine A2A receptor, GPR81/HCAR1, and SUCNR1/GPR91, respectively (Lee et al., 2018; Li et al., 2023; Zhang et al., 2024a; Dong et al., 2025). Inosine associated with *Akkermansia muciniphila* or *Bifidobacterium infantis* can regulate inflammatory responses, leptin sensitivity, and myocardial injury through the adenosine A2A receptor (Zhang et al., 2024a; Dong et al., 2025). Activation of GPR81/HCAR1 by lactate promotes Wnt/β-catenin signaling and the expansion of Lgr5^+^ intestinal stem cells, whereas succinate improves high-fat diet-induced intestinal barrier dysfunction and metabolic inflammation through SUCNR1/GPR91 (Lee et al., 2018; Li et al., 2023).

Biogenic amines further expand the range of GPCRs responsive to microbiota-derived metabolites by engaging neurotransmitter receptors. Histamine derived from *Lactobacillus reuteri* and microbiota-derived cadaverine act on H2R/H1R and H4R, respectively, and participate in the regulation of intestinal inflammation, inflammation-associated colon tumorigenesis, and sensory neuron excitability (Gao et al., 2015; Shi et al., 2019; Sun et al., 2024). Histamine suppresses TNBS-induced intestinal inflammation through H2R and produces a functional output opposite to that mediated by H1R with respect to MAPK activation and chemokine expression in inflammation-associated colon tumorigenesis (Gao et al., 2015; Shi et al., 2019). Cadaverine activates PLC-TRPV1 signaling through H4R, thereby enhancing the excitability of small-diameter sensory neurons in the dorsal root ganglia and inducing acute itch responses (Sun et al., 2024).

Overall, other microbiota-derived small molecules participate in the regulation of intestinal inflammation, barrier repair, immunometabolism, neural function, and distal organ function through multiple classes of GPCRs. Current studies have mainly focused on receptor-dependent and G protein-related signaling pathways, whereas direct evidence for β-arrestin remains limited. Future studies should further determine whether microbiota-derived ligands induce β-arrestin recruitment, receptor internalization, signaling scaffold formation, and biased signaling outputs through receptors including GPR119, GPR120/FFAR4, GPR84, SUCNR1/GPR91, P2Y10/P2Y10b, and H4R, thereby clarifying their roles in non-canonical GPCR signaling.

### 4.3 GPCR signaling and β-arrestin regulation by gut microbiota-derived peptides and proteins

#### 4.3.1 Host GPCR activation mediated by microbiota-derived peptides and proteins

Gut microbiota-derived peptides and proteins primarily activate host GPCRs through two mechanisms: short peptides directly bind to receptors, whereas microbiota-derived proteases cleave the extracellular region of receptors to expose tethered ligands (Wollam et al., 2019). The former is exemplified by formyl peptide receptors. The N-formyl peptide fMLF produced by the gut microbiota can activate FPR1, indicating that microbiota-derived short peptides can be directly recognized by host GPCRs. The latter mechanism is primarily observed for protease-activated receptors. GelE derived from *Enterococcus faecalis* increases intestinal epithelial permeability through PAR2, whereas Bfp1 derived from *Bacteroides fragilis* induces barrier damage and sensory neuron sensitization through PAR2 activation (Maharshak et al., 2015; Lakemeyer et al., 2025). In addition, serine protease activity derived from commensal bacteria can reduce sensory neuron excitability through PAR4, although the specific active protein has not yet been identified, and this evidence therefore remains conditional (Sessenwein et al., 2017).

These studies indicate that gut microbiota-derived peptides and proteins can directly participate in the regulation of host GPCRs. However, existing studies have focused primarily on receptor activation and biological effects, with relatively limited attention to signaling allocation and receptor trafficking following receptor activation.

#### 4.3.2 β-arrestin signaling regulation and mechanistic validation

Following GPCR activation, ligand-induced receptor conformational changes and GRK-mediated phosphorylation regulate β-arrestin recruitment, thereby influencing receptor desensitization, internalization, recycling, and intracellular signaling. Thus, in addition to modulating the magnitude of receptor activation, microbiota-derived peptides and proteins may alter signaling partitioning between G proteins and β-arrestins. However, whether such effects confer β-arrestin bias must be determined through quantitative comparisons across signaling pathways.

At present, evidence directly implicating gut microbiota-derived peptides or proteins in β-arrestin regulation remains limited. Studies involving non-gut microbial systems may offer mechanistic insights, but they cannot be considered direct evidence for gut microbiota-derived ligands. For example, gingipains secreted by the oral pathogen *Porphyromonas gingivalis* induce Src- and β-arrestin-dependent receptor internalization and ERK signaling via PAR2 (Nonaka and Nakanishi, 2020). Following MRGPRX2 activation, the bacterial quorum-sensing peptide CSP-1 and the host peptide PAMP-12 elicit different degrees of β-arrestin recruitment, receptor phosphorylation, and internalization (Amponnawarat et al., 2026). Meanwhile, *Staphylococcus aureus*-derived PSMα peptides activate FPR2-mediated Ca^2+^ and ERK signaling, but induce limited β-arrestin recruitment. Collectively, these studies indicate that receptor activation, β-arrestin recruitment, and downstream signaling are not interchangeable readouts and should therefore be evaluated independently using distinct experimental approaches (Sundqvist et al., 2019).

Gut microbiota may also indirectly influence β-arrestin pathways by modulating host peptide signaling. For example, microbiota-associated changes in bile acids can regulate postprandial GLP-1 release, while β-arrestin recruitment to GLP-1R contributes to receptor desensitization and the dynamic regulation of signaling (Jones et al., 2018; Wang et al., 2023). Such evidence reflects the indirect modulation of host peptide-GPCR signaling by the microbiota and should be distinguished from evidence of direct GPCR engagement by microbiota-derived peptides or proteins.

For gut microbiota-derived peptides and proteins, future studies should first establish their microbial origin and direct interactions with receptors, and then assess β-arrestin recruitment, receptor trafficking, and associated functional effects. GPCRome-scale screening can facilitate the identification of candidate receptors and interactions; however, mechanistic conclusions require further validation through orthogonal assays, causal perturbations, and comparisons with G-protein signaling pathways. Where feasible, these findings should also be further validated in human-derived and gut-relevant experimental systems.

### 4.4 Direct β-arrestin evidence across receptor systems

Current studies support links between microbiota-derived or microbiota-associated molecules and host GPCRs at the levels of receptor activation, ligand binding, and functional responses. Among these systems, TGR5, GPR35, and PAR2-, MRGPRX2-, and FPR2-related receptor systems have further received direct experimental support for β-arrestin involvement, including β-arrestin recruitment, receptor internalization, and β-arrestin-dependent scaffold or downstream signaling (Table 1). These findings indicate that β-arrestin has been directly implicated in the signaling mechanisms of selected microbiota-associated GPCR systems.

**Table 1:** Direct and indirect evidence relevant to β-arrestin involvement in microbiota-associated GPCR signaling.

Receptor	Ligand or ligand class	Evidence tier	β-arrestin assay	ARRB1/ARRB2 involvement	Experimental system	Species	Reference
FFAR2 (GPR43)	Microbial acetate	I	Not assessed	Not assessed	Gpr43 knockout mice with DSS colitis; human NCM460 and HT-29 cells; CHO-K1 cells transfected with GPR43	Mouse; human; hamster	Macia et al., 2015
HCA3 (GPR109B)	Indole-3-lactic acid (ILA)	II	PathHunter β-arrestin-2 recruitment	ARRB2 recruited in HEK293T cells; ARRB2 dependence of the monocyte response not tested	HEK293T cells transfected with HCA3; primary human monocytes with HCA3 siRNA	Human	Peters et al., 2020; Laursen et al., 2021
GPBAR1 (TGR5)	Ileal ωMCA and HCA (microbiota dependent)	I	Not assessed	Not assessed	Microbiota-depleted and TGR5 knockout mice; primary mouse ileal cells	Mouse	Wang et al., 2023
S1PR2	Signaling associated with DCA^†^	I	Not assessed	Not assessed	C57BL/6 J mouse indomethacin enteropathy; bEnd.3 mouse endothelial cells; Wistar rat thoracic duct lymphocytes	Mouse; rat	Shibuya et al., 2021
P2Y10	TDCA (microbiota dependent)	I	Not assessed	Not assessed	HEK293T cells overexpressing P2Y10 (Ca^2+^ and RhoA assays); P2ry10 knockout mice; intestinal ILCs; human intestinal tissue expression	Mouse; human	Xu et al., 2025a, 2025b

Tier I, receptor-associated functional evidence without a *β*-arrestin assay for the listed ligand or condition; Tier II, direct *β*-arrestin recruitment without evidence that ARRB1/ARRB2 causally mediates the listed physiological response. Tiers apply to the specific ligand-receptor-readout combination. ^†^S1PR2 involvement was inferred from partial inhibition by JTE-013; direct DCA-S1PR2 binding was not demonstrated. For HCA3, Peters et al. demonstrated ILA-induced ARRB2 recruitment in HEK293T cells, whereas Laursen et al. demonstrated an HCA3-dependent monocyte response without testing ARRB2 dependence.

ARRB, β-arrestin; DCA, deoxycholic acid; DSS, dextran sulfate sodium; HCA, hyocholic acid; ILA, indole-3-lactic acid; ILC, innate lymphoid cell; TDCA, taurodeoxycholic acid; ωMCA, omega-muricholic acid.

Importantly, the types and levels of β-arrestin evidence are not uniform across receptor systems. Receptor activation, β-arrestin recruitment, receptor internalization, and β-arrestin-dependent downstream signaling represent distinct experimental readouts and should not be considered interchangeable. Some PAR2-, MRGPRX2-, and FPR2-related studies also involve non-gut microbial ligands or comparative experimental systems and therefore primarily provide mechanistic context. Overall, direct β-arrestin involvement has been demonstrated in selected receptor systems, but the available evidence remains limited.

## 5 Discussion and perspectives

### 5.1 Summary

The gut microbiota can influence host metabolism, immunity, barrier function, and neural functions through diverse signaling molecules, including short-chain fatty acids, bile acids, tryptophan metabolites, other small molecules, and microbiota-derived peptides and proteins. These molecules can be produced directly by microorganisms or generated through microbial transformation or host–microbiota co-metabolism. GPCRs represent a major receptor system through which the host senses these signals and, together with nuclear receptors and other sensing pathways, constitute the gut microbiota-host signaling network.

Based on a literature survey of the near-complete non-olfactory GPCRome (314 conventional GPCRs), 137 receptors have varying degrees of evidence linking them to the gut microbiota, whereas only 56 receptors are supported by relatively well-defined evidence involving microbiota-derived small molecules, peptides, or proteins. Current studies remain predominantly focused on small molecules and a limited number of well-characterized GPCRs, whereas direct ligand evidence for microbiota-derived peptides, proteins, and orphan receptors remains relatively scarce. Most studies have also primarily focused on receptor involvement and functional phenotypes, while continuous validation of the direct roles of β-arrestin remains lacking.

Therefore, β-arrestin recruitment, β-arrestin dependence, and β-arrestin bias must be strictly distinguished. Current evidence suggests that β-arrestin may participate in the spatiotemporal regulation of GPCR signaling through receptor internalization, intracellular sorting, endosomal signaling, and scaffold functions; however, its scope of applicability and mechanistic boundaries remain to be further defined, and the available evidence is still insufficient to consider it a universal mechanism underlying all microbiota-GPCR interactions.

An additional limitation is that part of the available mechanistic evidence is derived fromrodent models or heterologous expression systems. Differences in receptor pharmacology andcellular context may affect ligand responsiveness, β-arrestin recruitment, and downstreamsignaling, and these findings should therefore be interpreted cautiously when extrapolated tohuman GPCR signaling and further validated in human-relevant systems where possible.

### 5.2 Perspectives

With advances in the identification of microbiota-derived molecules, GPCRome screening, and β-arrestin detection technologies, microbiota-GPCR research has reached a stage where it can transition from individual ligand–receptor discoveries toward systematic investigation. Future efforts should not be limited to expanding the number of candidate molecules and receptors, but should instead establish a continuous pipeline extending from ligand discovery to mechanistic determination and physiological validation, allowing evidence obtained at different levels to be effectively integrated.

This transition first requires systematic organization and gradual refinement of the ligand library derived from microbial communities. The ligand repertoire should encompass short-chain fatty acids, bile acids, tryptophan metabolites, other small molecules, as well as microbiota-derived peptides and proteins, while defining their origins, structural information, and physiological relevance whenever possible. A standardized ligand resource can provide a unified input for GPCRome screening and may also help reduce long-term dependence on a limited number of well-characterized receptors.

On this basis, GPCRome platforms can expand the discovery scope of candidate ligand-receptor relationships. Tango and PRESTO-Tango can be applied for initial screening and provide indications of β-arrestin recruitment; however, positive recruitment signals cannot directly demonstrate β-arrestin dependence or bias, and further validation with G protein pathways and other orthogonal approaches is still required.

High-throughput screening is responsible for identifying candidate relationships, whereas mechanistic studies are required to determine whether these relationships are truly established. β-arrestin dependence should be confirmed through genetic causal interventions, whereas β-arrestin bias requires quantitative comparison with corresponding G protein pathways under standardized conditions. Furthermore, integration of analyses including receptor internalization, intracellular sorting, endosomal signaling, subcellular localization, and signaling duration is required to determine whether candidate ligands alter the temporal, spatial, and functional allocation of GPCR signaling.

These efforts may provide a basis for establishing and testing models of the role of β-arrestin in the spatiotemporal reprogramming of GPCR signaling and for identifying key microbiota-derived ligands, host GPCRs, and potential regulatory targets.
